# A Matter of Taste? Quality of Life in Day-to-Day Living with ALS and a Feeding Tube

**DOI:** 10.1007/s11013-015-9479-y

**Published:** 2015-11-07

**Authors:** Jeannette Pols, Sarah Limburg

**Affiliations:** Department of General Practice, Section of Medical Ethics, Academic Medical Centre, Postbus 22700, 1100 DE Amsterdam, The Netherlands; Department of Anthropology, University of Amsterdam, Amsterdam, The Netherlands

**Keywords:** Quality of life, Ethnography, Daily life, ALS, Feeding tube, Research methodology

## Abstract

Although people often refer to quality of life and there is a respectable research tradition to establish it, the meaning of the term is unclear. In this article we qualitatively study an intervention of which the quantitative effects are documented as indecisive. We do this in order to learn more about what the meaning of the term quality of life means when it is studied in daily life. With the help of these findings we reflect on the intricacies of objectifying and measuring quality of life using quantitative research designs. Our case is the feeding tube for patients suffering from ALS, a severe motor neuron disease that rapidly and progressively incapacitates patients. We studied how these patients, who lived in the Netherlands, anticipated and lived with a feeding tube in the course of their physical deterioration. Our analysis shows that the quality of life related to the feeding tube has to be understood as a process rather than as an outcome. The feeding tube becomes a different thing as patients move through the various phases of their illness, due to changes in their condition, living circumstances, and concerns and values. There are very different appreciations of the way the feeding tube changes the body’s appearance and feel. Some patients refuse it because they feel it disfigures their body, whereas others are indifferent to its appearance. Our conclusion is that these differences are difficult to grasp with a quantitative study designs because ‘matters of taste’ and values are not distributed in a population in the same ways as physiological responses to medication. Effect studies assume physiological responses to be more or less the same for everyone, with only gradual differences. Our analysis of quality in daily life, however, shows that what a treatment comes to be and how it is valued shows shows generalities for subgroups rather than populations.

## Introduction

The meaning of ‘quality of life’ is not well understood. The interest in quality of life started as a way to include patients’ subjective evaluations in research on the effects of treatment, besides the usual medical outcome variables. The idea was to incorporate the possibility that while a particular therapy may solve a medically defined problem (‘treatment X extends life by three months on average’) the patient does not actually benefit (‘I live in a permanent state of fatigue and cannot work anymore’). In this paper we are interested in the measurement of quality of life as a mode of quantification made in clinical trials and comparable designs. Quality of life is then shaped as an outcome variable that is assessed through questionnaires. Scores are aggregated for a particular group of patients, and signify the effect of a treatment on a certain population.

What is measured when quality of life is measured? The WHOQOL Group (1995) describes it as “individuals’ perception of their position in life in the context of the culture and value systems in which they live and in relation to their goals, expectations, standards and concerns.” The description shows the recurrent problem of measuring quality of life as a subjective evaluation (‘individuals’ perception’) and/ or as a condition that is evaluated from an external point of view (‘culture and values systems’). Consequently, some researchers assess quality of life by asking people for an overall rating of happiness, that is, the grade they would give to evaluate their life (Pais-Ribero 2004). Yet other measurements, such as the EuroQol (EuroQol Group 2009) do not contain individual assessments, but establish how well patients are functioning (‘I have no problems in walking about’). Sometimes mental health is measured as an indicator for quality (e.g. the Beck Depression Inventory), and there are questionnaires for different forms of cancer, stroke, ALS and so on, some used in research, others to evaluate patients’ clinical situation. Apart from these there are the QALY’s and DALYs that are used in international studies that asses the ‘Global Burden of Disease’ (see Moreira 2012). These studies inform the funding and accessibility of treatments and shape health care policy globally. Apart from these very different assessments, there are many disease-specific questionnaires on quality of life. However, the contents of these questionnaires are often not described in detail in the method sections of journal articles. Often we simply do not know how the researchers operationalized quality of life.

All in all, the measurement of quality of life has a huge impact on policy and treatment, but there is unclarity and dissensus on what quality of life should mean. This makes some authors utterly cynical about its measurement. Quality of life measurements, they argue, have become strategic measurements demanded by public and private sector initiatives, turning them into means for selling treatments rather than serious assessments (Hunt 1997a, b). Questionnaires that claim to measure quality of life are tagged onto clinical trials, where ‘quality’ or patients’ experiences of quality are hard to find (Carr-Hill 1991; Carr and Higginson 2001).

The lack of insight into what quality-of-life questionnaires evaluate also leaves certain observable facts such as the ‘disability paradox’ unaccounted for. The ‘disability paradox’ refers to the phenomenon that people with severe handicaps or diseases often report a good, stable or even improved quality of life, while other people imagine that they would be very concerned about their disability (Albrecht and DeVlieger 1999; Lacey et al. 2011; Gauthier et al. 2007). More semantic confusion emerges when *changes* in quality-of-life measurements are interpreted, as it is unclear whether these refer to actual changes in quality in real life or to measurement errors. This fuelled the debate on ‘response shift’ (Sprangers and Schwartz 2010; Ubel et al. 2010; Eton 2010), a term that refers to ratings of the same phenomenon (e.g. being in a wheelchair) shifting over time.

We sympathise with the idea that it is important to find ways to include patients’ experiences and values in the evaluation of medical treatments. We are, however, concerned about the way research into quality has developed. Although the global implementation and impact of quality of life measurements on health policy and treatment is enormous, we dare to re-open the question about the meaning of quality of life, to see how its measurement relates to patients’ values, anticipations and evaluations of treatment.^1^ Our aim is not to debunk quality of life measurements, but to explore what these measurements can make visible or not, and how (and if) they relate to a practical understanding of quality in everyday life.

We start from the suggestion that much confusion on what quality of life might mean emerges precisely because of the specific way the object ‘quality of life’ is articulated through its assessment (measuring) and its specific mode of quantification. This involves particular assumptions about what quality is and how it is spread in a population. In order to learn about the meaning of quality of life we shift the focus away from the scientists deliberating about quality of life, and towards the clinic and to people living with a severe and progressive chronic disease.^2^ Our aim is to analyse what quality of life comes to mean by studying it in the daily lives of patients. To do this we conducted a theoretically informed qualitative empirical study. How may we understand quality of life as part of everyday life? In the conclusions we will return to the question how an understanding of quality as part of daily life sheds light on attempts to measure and quantify it.

### The Case

Our case looks at the lives of people in the Netherlands suffering from ALS (amyotrophic lateral sclerosis) who are considering obtaining a feeding tube, or who have been living with a feeding tube for some time. ALS is a severe progressive motor neuron disease. Because of the degeneration of nerve tissue that instructs the voluntary muscles, patients are progressively unable to move and the muscles waste away. The course of the disease is generally devastating: 50 % of patients die from ALS within 3 years of diagnosis; most patients are dead 5 years after.

A feeding tube (gastrostomy) involves piercing the stomach wall to insert a plastic tube into the stomach. Fluid nutrition can be fed through the tube, either manually with a syringe, or through a motor-propelled drip. There are several methods for tube placement (see Stavroulakis et al. 2013 for a clear description). In the hospital where we did the study, percutaneous endoscopic gastrostomy (PEG) was the most common procedure, as it is elsewhere. With PEG insertion, the patient has to swallow a scope that illuminates the stomach from within and the stomach wall is pierced from the inside out, thereby minimizing potential damage to blood vessels. PEG can only be performed when patients have sufficient lung capacity and do not depend on breathing devices. PEG placement is done by a specialist, the gastroenterologist, who in our study was associated with the ALS team and knew the patients from earlier consultations on ways of dealing with dysphagia (swallowing problems due to the weakness of the tongue and mastication muscles). When a patient does not meet the requirements for PEG but can lie on their back, the radiologist inserts the tube: radiologically inserted gastrostomy (RIG). With RIG insertion, the stomach is inflated with air and the stomach wall is pierced from the outside in. The diameter of the tube is smaller than for PEG, fixed less stably, and the wound needs to be stitched, increasing the risk of infection. The radiologist does not know the patient and this is one reason why PEG is preferred in the hospital where we conducted our study. The reasoning here is that as talking becomes difficult for the patient, and their body has lost much of its strength, a familiar doctor enhances communication and feelings of safety and trust.

### Quality of Life and the Feeding Tube

There are various reasons for considering the placement of a tube, including excessive weight loss through dysphagia, difficulty with coughing that may result in (an acute fear of) choking and the risk of getting pneumonia, and excessive time spent on feeding. The literature on the effects of tube feeding on the lives of patients with ALS is inconclusive. The big questions in this literature are whether tube feeding extends life and improves nutritional status, and whether and how it interferes with quality of life. There is no solid statistical evidence for either of these effects (Benatar and Katzberg 2011; Langmore et al. 2006).

In terms of the feeding tube, good quality of life is mostly related to the social event of eating (Vesey et al. 2008), whereas tube feeding is related to physiological effects such as obtaining enough calories, longer survival and improved nutritional status (Goyal and Mozaffar 2014; Shintani 2013; Greenwood 2013). Some authors warn that withdrawing the possibility of eating through the mouth should be last resort because of the many meanings attached to eating (Vesey 2013; Aparanji and Dharmarajan 2010; Todd et al. 2005). Other authors, however, recognize the problem of having a all-day job in trying to swallow food (Martin et al. 2012), and see tube placement as a minor surgical procedure (ibid) or a minimally invasive procedure (Hossein et al. 2011) that will enhance quality of life (Mazzini et al. 1995).

Because there is no clear evidence for the effects on either quality or survival, some authors argue that the decision to insert a feeding tube should only be made if improvement of quality or life is to be expected, as opposed to ‘prolonging the dying process’ (Todd et al. 2004; Angus and Burakoff 2003; Pennington et al. 2002). This may be hard to determine, and patients, their loved ones and clinicians may all take a different stance towards it (see Kaufman 2015). Others authors argue, however, that dysphagia itself may be a deficit in quality of life, and that the physiological benefit of a persons’ nutritional status should be a necessary condition for placing a feeding tube (Rabeneck et al. 1997). Our study comes at a time when confusion rules and one might ask whether we should conduct a solid trial to measure the effects of tube feeding on quality of life (and survival), and if so, what would the outcome tell us. Our study makes a case to assess this question.

## The Study

### The Informants

For our study we (SL & JP) interviewed patients whom we met through the ALS Tertiary Care Centre in the academic hospital in the urbanized region of the Netherlands, through the ALS Stichting Nederland (a national funding agency for research into ALS), through social media and through personal connections. The theoretical design of the study aimed to find patients in different stages of relating to the feeding tube, varying from anticipation to experienced use. We interviewed 11 ALS patients anticipating (3) or living with a tube (8) or at both times (2). In total we recorded and/or transcribed 15 new interviews for this study.

We also used observation and interview material our colleague collected for her study on advanced care planning (Seeber 2014, in preparation), which gave us extra material on 28 people with ALS who she had followed over at least three consultations. The observations early on in the trajectory, when patients heard their diagnosis, were an especially welcome addition, as tube feeding was usually only considered at a later stage. These allowed us to get an overview of the whole trajectory and its variations. When we use this material we refer to the author, the unmarked material is from our new interviews and observations.

Interviewing ALS patients took particular patience from both the interviewed and the interviewer. Due to muscle weakening, talking could be difficult for informants. Partners and children supported them by explaining things to the interviewer. Some informants used a speech computer. Others could not speak at all, and delivered their story to us in writing or through the spouse. According to Dutch law and research codes we did not need the approval of an ethical committee for this study, but with this particularly vulnerable patient group we took special care to let people know they could always opt out of an interview or cancel an appointment, which they sometimes did.

We also interviewed the professionals concerned with tube feeding in our hospital at different points in time: the gastroenterologist, the neurologist concerned with diagnosing ALS patients, the nurse specialized in coaching patients with feeding tubes, and the rehabilitation doctor, who was the central carer for ALS patients. He helped us to approach patients by handing out our information letter to them. If patients wanted to participate voluntarily, they could tell the doctor or nurse, who would then give us their contact details. We discussed our results with the rehabilitation doctor to check our findings and interpretations.

Besides interviews, we observed three consultations of patients and their partners who came in to discuss tube feeding with the gastroenterologist, and two consultations with the specialized nurse. This is how we learned what the specialist told patients about tube placement and living with a feeding tube. When patients arrived here, they already had discussed the consequences of tube feeding with the rehabilitation doctor. Due to the ‘lack of clear evidence’, his approach was tailored to the specific situation of the patient, who ultimately had to decide for or against having the tube. When possible, we observed patients using the feeding tube, or asked them detailed questions about using it, turning the patients into ethnographers of their own situation.

### Methodology

This research builds on earlier studies in empirical ethics into care practices related to the concept of dignity (Pols 2013a, b). Empirical ethics ethnographically studies ‘normativity in practice’ (Pols 2013a, b, Pols 2015; Willems and Pols 2010; Mol 2010; Thygesen and Moser 2010). Normativity can take different empirical shapes, and refers to (attempts to) do what is ‘good’, where what is ‘good’ is not given but has to be established empirically. There may be norms to follow, values to put into practice, ideals to strive for, tastes to appreciate, judgements made or prescriptions implemented. From the exploration of what dignity might mean in care we learnt that it is helpful to distinguish between ethical and aesthetic values. With ethical values we refer to principles that are valid for everyone, everywhere. An example is patient autonomy that should be respected everywhere. On the other hand, aesthetic values show the differences in what people value—proverbially, different people have different tastes.

This does not mean that preferences are products of authentic individual desires; these are embedded in a wider social-cultural context. Importantly, aesthetic values may have more or less moral weight. It was the combination of situated, relational and contingent nature of aesthetic values related to dignity that together with their heartfelt moral weight made them hard to deal with in common ethical repertoires. The category of aesthetic values opens up matters of what is stylish, tasteful, embarrassing, degrading, or ‘of good quality’. We take quality of life as a broader value than dignity, as it may relate to aesthetic values that carry less moral weight than dignity does. The importance of, say, that nice cup of tea every morning, or the joy of hearing one’s favourite music may be crucial to a particular person’s appreciation of life, but it would not easily stir moral controversy. Aesthetic values that are part of what gives life its quality may thus also concern the less normative and less socially enforced appreciations people might have.

The distinction between ethical principles and aesthetic values is relevant because, just like statistical methods, each approach deals with differences between people and situations in particular ways. Principle ethics looks for general values that count for everyone. Morally speaking, individual peculiarities are irrelevant as the principles articulate what people have in common (autonomy, freedom, equality for the law). Rawls’ veil of ignorance is meant to erase individual differences in position and status from just decision-making. Statistical calculations are also ways to articulate general tendencies, this time not in terms of universal laws, but in terms of generalizable probable effects of interventions on populations. Again, individual differences are mostly trivial—a person’s appreciation of expressionist painting is irrelevant to the establishment of treatment effects. General effects (e.g., fatigue) can be made visible, but differences between people on variables other than those defined as outcome variables disappear from the average. Aesthetic values or matters of taste differ for different people, and are not necessarily divided following a Gaussian curve in a population of patients. Therefore, by articulating them we create space in our analysis to see how these differences play out in the patients’ assessment of the feeding tube, and how these assessments would relate to general assessments of quality of life.

To anchor our interest in the differences between people in our methodology, we distinguish between (anticipated) *changes* in life related to tube feeding and the way patients *value* these changes. We analyse quality as either a *normative* or a *descriptive* category. The normative meaning of quality makes the word stand for something good, like a talent, a virtue, a preferred activity or a capacity. Colloquially this meaning of quality is used in sentences like: ‘I opt for quality of life rather than treatment’. Quality is then good in itself, it expresses value. This is the way quality of life is interpreted in quantitative research, measuring more or less or average, ‘overall’ or ‘health-related’ goodness.

The second meaning is descriptive, as in ‘qualitative research’ that describes a type of research rather than praises it. In this descriptive sense, quality refers to a characteristic or property that has yet to be evaluated. When quality is a characteristic, the question if a certain treatment leads to more or less quality of life is meaningless; it would translate as the question whether life with or without a treatment has more or fewer characteristics. It is, however, relevant that a treatment may lead to different characteristics of life for different people. So our distinction forms an analytical sensitivity to learn how and where characteristics and their valuations hang together, where they differ, and where it is useful to discern them or not.

#### Research Questions

Our questions are: What kind of intervention is the feeding tube? What characteristics and appreciations does anticipating or living with a feeding tube bring to ALS patients’ daily lives, and (how) does this differ between patients and situations? What does it teach us about the meaning of quality-of-life measurements as a method for evaluating the feeding tube?

## Results

### The Enigmatic Character of the Feeding Tube: The Hospital

In the hospital where we conducted our research, ALS patients clearly tend to postpone the decision on taking a feeding tube as long as they possibly can; this fact is also reflected in the literature (e.g. Stavroulakis et al. 2013),. The gastroenterologist preferred inserting tubes earlier, when patients were relatively fit and could benefit from the nutrition and not having to spend all day trying ingest food. The gastroenterologist was not sure that the patients fully understood what getting and living with a feeding tube actually entailed, as many of those who opted for late placement died within 3 months of treatment.

The concern about postponing tube placement was strengthened by a failed trial in the neurology department (Van der Graaff, in preparation 2014). The researchers wanted to compare two conditions. In the first condition, patients decided when to get a feeding tube; in the second the doctors decided the timing. The trial was never conducted because patients did not want to enrol. The reasons they gave for refusing to participate were mostly along the lines of ‘they were not ready for a feeding tube yet’ and had ‘enough on their plate’. They did not want to run the risk of being assigned to the condition where the doctor would decide. The trial failed, but this failure is significant as it shows the emotional weight patients attach to the placement of the feeding tube, and their reluctance to have it. The decision to take a feeding tube is complex. In our study we systematically mapped these complexities by following the trajectory of patients anticipating a feeding tube, and their life with it after they obtained it, at various points in time. We now show how the identity of the feeding tube changed in the course of patients’ trajectory.

## Characteristics and Appreciations Change Together

### From Symbol of Deterioration to Eraser of Complaints and Concerns

When they receive their diagnosis, the ALS patients has to face a radically new perspective on life and a very concrete view of their impending death. Seeber et al. 2014 show that the diagnosis is a shock from which most patients have to recover before they can reorganize their lives. They usually start at the end: they anticipate how they will die and discuss their wishes for care at the end of life with their GP (ibid). The trajectories are different for different patients, and the feeding tube is one of many issues that they have to come to terms with.

When they do not suffer from dysphagia yet and there is no problem with weight loss, the patients in our study had tube feeding on their list as something they *might* need later. But this ‘later’ was characterized by an image of decline in ways they would rather not think about if they could avoid it. At this point, the feeding tube was a scary *symbol of deterioration* or even an imagined limit to a life worth living. Patient Piet looks back:

You see, that’s another change of horizon. When it all started I said: when I get to the point where I need a [feeding] tube, really, that’s when I don’t want to go on. But then I never wanted to be in a wheelchair. And now I say to my wife: “Come on, give me a push, let’s go out together!” So we’ll have to see… (Seeber, unpublished material)

The patients took the progression of their disease one step at a time. What added to the negative identity of the feeding tube was that the patients found the *procedure* of placing the feeding tube terrible. The literature describes tube placement as a minor surgical procedure (Martin et al. 2012), or a minimally invasive procedure (Hossein et al. 2011). This may be true for the doctors, and comparable examples have been reported where physicians downplay the effects of their treatment.^3^ In our case, however, without exception patients agreed on the gruesomeness of the tube placement procedure. They anticipated the placement fearfully and afterwards reported on the horror of being put in an awkward, powerless physical position, finding it hard to breath and having to swallow the scope, which sometimes caused spasticity and blocked the larynx. The procedure demanded hospitalization, which implied a displacement from where the patients had their support and technological adaptations. The weaker the body, the harder the placement procedure was for them. There was a remarkable consensus among patients in their evaluation of tube placement, with the main variations mentioning just how terrible it had been, even if the doctor thought the procedure had gone smoothly and quickly.

Although some patients said that their own opinion was not of primary importance in the decision to get a feeding tube (“I had no choice…”; see also Vesey 2008), for other patients, it was. Typically, these patients’ narratives, like Piet’s quoted above, showed a turning point. The identity of the feeding tube changed.

Interviewer: Can you tell me what happened to make you need a feeding tube?

Mr Jansen: I have problems swallowing, and I choke. And eating takes a very long time… One month, two months ago, eating dinner took the whole evening. I can’t swallow food. So that’s why, really. […] And then it takes you more than an hour to eat. It takes so much energy. And you leave lots of food on your plate, because you give up trying. So then I lost weight, I was underfed. And I lost more and more weight. So at a certain moment… We have a very good doctor and she wanted to do it [the placement] before she retired. So we had to think about it a lot, before we accepted the feeding tube.

Interviewer: What did you have to think about?

Mr Jansen: Well, of course, the fact that you have such a thing in your stomach wall!

Mrs Jansen: Yes, you considered the down side, eh? But then, this explanation [by the doctor], that was really nice. She told us everything about it. And then we knew it just had to be done. We [the family] decided immediately. But Hans [Jansen] said: I don’t want it. So we took a leaflet home, deliberated, considered. That was Wednesday. And then, the other day, [to husband:] you choked terribly, [to interviewer:] he chokes every day, but this time we thought: “this is the end”. And the boys [sons] were there and we said to Hans: “What do you want? Do you want to choke?” And then he said: “You’ve convinced me.” And he sent an email on Friday, straight away. [to Hans:] And you even looked forward to it!

The idea of a feeding tube changed for Mr Jansen when he could see that the tube would not only be awkward and disfigure him, but it would provide things that were of value to him. For him it meant no more choking, no more fear of choking to death, no more struggling to eat, which no longer tasted so good anyway, and no more weight loss. As his wife reported, the prospect of leaving all this behind was something Mr Jansen actually started to look forward to. The feeding tube turned from a symbol of deterioration into a means towards an end: it became an *eraser of complaints and concerns*.

This is an example of how the characteristics of life with a feeding tube and the value of these characteristics intertwine and change together. In every-day life, the feeding tube became a different object. The tube now provides valuable opportunities rather than solutions to problems Jansen did not experience. These possibilities outweighed his primary, self-evident reluctance to ‘have something in your stomach wall’. This was not something he would ever consider for trivial reasons. The extremely scary event of nearly choking to death, however, was the tipping point to realising that a tube could be applied for good reasons. This shift not only relates to a *psychological* process or change in perceptions, as in response shift, but to a process of shifting physical qualities. When regarding the shift in these terms, the tube is a rational response to a changed situation rather than an inexplicable change in values.

It is also possible that only the patient’s opinion changed, even if the situation didn’t. Mrs Geerts explains her husband’s lack of interest in food.

Some things take care of themselves, things you can’t do any more, like going out. I remember I couldn’t ride my bike. At a certain point you start hating cycling because it’s so hard to do, you can’t get on or off the bike, you see. So then it’s not a problem that you can’t cycle. It’s like that: things are just not possible at a certain point. Then the need disappears, even if you’ve always liked getting out. We travelled a lot, we were always out and about. You can’t do that any more, and now it would really bother me if I had to pack my suitcase, so to speak. You can compare it to that!

This change in preference is often called a ‘response shift’, which is defined as involving ‘changing internal standards, values and the conceptualization of quality of life’ (Sprangers and Schwartz 1999). The phenomenon to be explained is a change in the way people answer questionnaires, or as Eton (2010) says: “[R]esponse shift is […] a theory that helps us understand how certain psycho-social processes can affect how people answer questions on health-status measures (p. 930)”. It is also described as cognitive resonance reduction by psychologists looking for patterns in human behaviour; the same phenomenon (eating, cycling) is judged differently. We did not observe this phenomenon in anticipations about the placement of the feeding tube. Only when the feeding tube could actually solve problems would people change their mind about it.

The shift in the situation, to when choking becomes a real concern, makes it understandable why people postpone taking a feeding tube. It is not because they fail to understand how the tube works or value it in strange ways, but because it does not solve real problems in a life already full of difficult medical issues. If a feeding tube only solves problems that *might* occur at a later stage, people postpone this radical treatment in the hope they will never need it. The time to face it is when dysphagia does become a severe problem. Consequently, these people opted for tube placement very late in their trajectory, which made the procedure harder to bear, while the gains of being tube-fed were less (see below).

### The Tube as Facilitator of Happy Events and as Transformer of Misery

The identity of the feeding tube as eraser of complaints changed again. When people evaluated the feeding tube positively, the tube became either an eraser of complaints and concerns, or a *facilitator of happy events.* The latter happened when patients discovered that the time formerly spent on eating could now be spent on meaningful things. The patients described various happy events the feeding tube facilitated. Paradoxically, and different from the juxtaposition between the pleasure of eating and the necessity of tube feeding suggested in the medical literature, the tube permitted new ways of enjoying food and eating. Eating and tube feeding are not mutually exclusive, which is a common and sorry misunderstanding. Once the pressure was lifted to obtain enough calories, eating – or rather *tasting* – could be organized in new, enjoyable ways, albeit in another form. Eating had to be reshaped, and related to food that is not too liquid, or too fragmented, yet tasted good. It did not have to be ‘healthy’ or ‘fresh’ food, which puzzled some patients and their carers. Good taste, swallow-able food and pleasure were what mattered. One patient ate one or two cookies a day for the joy of *chewing*.

Partner: Eating was very difficult, and it took a lot of time.

Jenita: I was busy eating the whole day.

Partner: And obsessively, eh, because it is also a fight against losing weight. It was really tense. And now, with the feeding tube, she wins lots of time and energy that does not go into eating and worrying about food. She eats soup, custard, whipped cream, all the things she really likes. And it’s no longer the main thing, or a necessity.

For Jenita, eating had completely lost its attraction because of the difficulty she had with swallowing. With the feeding tube in place, the calories were taken care of, and Jenita could engage in eating in ways she liked. The substance of the food narrowed the possibilities (not everything can be swallowed easily, and not every taste can be made swallow-able), but within these restrictions she could eat the things she enjoyed in a relaxed way. Eating could become a pleasurable activity rather than a necessity to survive.

Mrs Velds: You really like yoghurt, Greek yoghurt. And we put fruit in it, a mashed banana, that’s what we did.

Mr Velds: Or a *mergpijpje* [a meringue coated in marzipan and chocolate] every now and then.

Mrs Velds: [laughs] Yes, a mergpijpje, something sweet, he likes that. Its soft.

Mr Velds: And yesterday we had chicken tandoori.

Mrs Velds: Yes, just for the taste. We mashed it, really crushed it because the bits of it are really hard. But he likes it anyway, so we put a little on his plate with something.

Mr Velds: With asparagus, yes. [laughs]

Interviewer: Also mashed?

Mrs Velds: Yes, yes! […] And now and then an alcohol-free lager, or just a beer. Like yesterday.

Mr Velds: Two! [smiles broadly. Mrs Velds and interviewer laugh too]

Clearly the couple had great pleasure in helping Mr Velds re-enjoy the taste of food. Disconnected from the need for calories and food intake, they made tasting food ‘choke proof’. One patient we interviewed, who was treated in peripheral clinics rather than an ALS centre, was not informed that she could ‘eat’ in both ways [with or without the tube]. This meant she lost the opportunity to gain pleasurable experiences, as she did not use the tube until she ‘really needed it’ and had to give up eating.

Besides the food lovers, there were also patients who had never found eating much fun. To them, the feeding tube meant ‘good riddance’ to an exhausting task. They had ‘never been big eaters’ anyway and could do very well without the hassle of daily meals. They would rather spend their time on other things.

Gastroenterologist: The tube does not give quality of life in the sense that it cures a patient, because they cannot be cured. The only quality it gives is that people say that they did not enjoy the social aspects of eating so much, ‘because it takes me hours to eat, my food gets cold’. Well, we give them plate-warmers, we do everything possible to facilitate eating. But at a certain point people say: ‘Oh, I’m so tired of it.’ I had one patient, he was an artist, a painter, who because of his ALS could only draw dots. He’d go to the zoo, and he’d would make dots with his pencil, make drawings just out of dots. And he said to me: “Thanks to the tube I have won so many hours in a day. I have only a couple of hours in the day when I am not too exhausted to draw my dots. Before, I used those hours for eating, and now I don’t have to do that anymore!”

The example shows that the feeding tube might allow for positive characteristics that do not in themselves relate to the tube, but *are facilitated by it*. So when the tube is not a positive characteristic *in itself*, it is important that patients consider what they could do with the time won by obtaining a tube.

The tube became a *transformer of misery* that swapped one form of unhappiness for another. This happened for people who could not administer their own tube feeding due to lack of muscle power and had no informal carers to do it for them. Particularly when they needed regular feeding (e.g., every two hours), the feeding tube could be a major source of misery or loss of goodness.

James: You’re right, I don’t spend hours stuffing myself with food anymore… Still, my daily life has not improved. It’s even worse as I am stuck at home now, waiting for the nurses to help me with the feeding bags (Seeber 2012).

James had to wait for professional carers on their erratic schedules to put the bags of artificial food on the motor-propelled drip he was on. Here the feeding tube gets its particular identity by the way it shapes and is shaped in the daily lives of patients. The tube does not function ‘by itself’, as a thing with relatively autonomous qualities and workings. It obtains its identity ultimately in the circumstances in which it is put to use, facilitating some things and making other things harder.

## Differing Appreciations

### Transformation of Misery Versus the Facts of Life

We have seen how the characteristics of both an appreciation of the tube and living with it changed together. But some valuations of similar events differed remarkably among patients. This became obvious when it concerned the valuation of “having a plastic tube sticking out of one’s belly”. Some patients had decisive reasons for objecting to that.

Interviewer: So it’s preventive, but you’d like to postpone it as long as possible.

Harry: Yes, obviously, it’s terrible, having a plastic tube coming out of your tummy.

Interviewer: What exactly is so bad about that idea?

Harry: Well, it’s plastic, where you don’t expect it. Or want it.

Lene [spouse]: Like you all [ethicists] say, it damages your physical integrity [the meaning of integrity refers simultaneously to the wholeness/ intactness of the body and to its violation, JP].

Harry: The first of the invasive things is the breathing device. But that’s from the outside. And if you take off the mask, after ten minutes it’s as if you never wore it. But the tube will always be there [you cannot take it off]. And unfortunately you can get a button [a flat plastic disc that closes the tube and hides the hole in the stomach. It demands agility to remove it and connect the syringe to an extension of the tube, but it produces a better aesthetic effect than the roll of plastic tubing taped to the patient’s trunk] only after three months. Honestly, I thought that I could have it [the button] right away. So that’s a bit of a disappointment.

The reluctance to have a tube by its relation to the sensual qualities of the body surprised the gastroenterologist, who saw maintaining physical fitness and longer survival as the ultimate goals, and the feeding tube as a means towards this end (“It’s your life-line!”). The more social, erotic and aesthetic meanings of the body were hard for the doctors to imagine in the face of issues of life, and death, and solvable problems. The gastroenterologist said in an interview that she had had the same experience with patients who had fallen victim to the first AIDS epidemic; they all wanted a button, caring about the way their body looked and how they would sense it. The doctor found this hard to understand in the face of death, but it points to the more sensual ways in which people use and live their body. Having one’s bodily integrity broken by a disfiguring tube—made out of a material that jars with the tissue which bodies are made of and that protrudes for others to see—could be a major problem. The rehabilitation doctor confirmed that some people reject the placement of a feeding tube for this reason. They rather live and die without the tube (see also Sakellariou 2013). Experienced PEG-users hid the tube with a sash or band of cloth around the body, so it would not show when a person was naked or in a swim suit. This is not an aid commonly suggested to people with ALS, but was a creative way to deal with concerns about how the body looked.

These sensual concerns with the feeding tube were very important and heartfelt for some, yet others were completely indifferent to it.

Interviewer: What did you think about having the tube sticking out?

Jenita: [Waves hand dismissively]. It’s not important. The benefits are. It’s not that I particularly like it; but it’s not an issue.

Obviously the feeding tube is not the same thing in Harry’s or Jenita’s lives. Again, something happens to the identity of the feeding tube. Yet this time it is not due to the characteristics of the environment in which the tube gains its function, but through a different sensual appreciation. For those who value the sensuality and appearance of their body highly, the feeding tube would be a transformer of misery. Where once they suffered dysphagia, fear of choking and weight loss, they now suffer from having a deformed body. For those who cared less about this type of aesthetics, the tube did not add to their suffering.

Jenita’s indifference to these concerns reveals something else, too. Apparently, people take some qualities as ‘facts of life’ and accept them as givens. These characteristics come with the feeding tube, but Jenita does not judge them as relevant to the value of her life. Something comparable seems to be at stake with the disability paradox. People do not judge their life as a handicapped person in comparison to a life *without* impediments. They have become a fact of life. They form the ‘givens’ after which everything else follows. Rather than “balancing positive and negative qualities”, as Albrecht and DeVlieger (1999) suggest, qualities shift in the way they are valued. When the researchers asked people about quality of life, they would ask themselves whether there were matters they enjoyed or found of value.

Mrs Ralphs: I just had my birthday. And they [her three kids] put on a heart-warming party for their Mum. And my mind is clear. There are many days with a silver lining.

Mrs Ralphs can no longer get out of bed, but she sees her life as valuable because of the events and characteristics she values positively. Rather than dwelling on what she has lost, she lists the positive things in her life. Disease is not in the calculation, *unless* there are symptoms such as nausea and fatigue that hinder her ability to experience the good things. These pervasive symptoms are the negative quality judgments that Albrecht and DeVlieger describe. Rather than being in balance, they overrule the possibility for enjoying the positive.

That a new condition of the body becomes a given is also visible in research into the experiences and training of people with other chronic diseases or disabilities. For example, studies of COPD patients show how ‘accepting’ their new conditions and (im)possibilities formed an important theme in their rehabilitation (Pols 2011). If they did not redefine their horizon of physical possibilities, they would keep running into trouble. The opposite pitfall was the fatalistic assumption that any attempt to improve their situation would be useless (Habraken et al 2008). The job of these chronic patients was to learn to live with their incurable disease rather than hope for its disappearance. This is a typical assignment for people with chronic illnesses. They have to live with ‘changed givens’, and try not to let these things erase what is of value in their lives.

## Discussion

In our quest for quality related to the feeding tube in daily life, it turned out the feeding tube had different identities. The feeding tube changed character depending on the shifting situation of the individual and played an active part in establishing the various situations. At first, the feeding tube began as a frightening symbol of deterioration, linked to gruesome placement procedures and hospitalization. The tube’s bad image changed when it became an eraser of complaints and concerns, offering a solution to problems patients actually experienced. This change in identity made the timing of the placement so difficult. Often the best time for the placement procedure is when the body is relatively fit, so that it might benefit from good nutrition and is resilient enough to deal well with the surgery. However, at this stage the tube has its negative symbolic identity. This was not the case for patients who had dysphagia without suffering other symptoms (bulbar onset). To them, the tube provided a solution relatively early in their trajectory. However, patients without dysphagia but suffering from paralysis of the limbs (limb onset) had to anticipate whether the tube could be of use to them *in the future*, at a point when they regarded the tube as a sign of a very bad life rather than a solution to a problem.

The tube did not keep its identity as an eraser of complaints and concerns. One step further in the trajectory the tube became either a facilitator of positive activities or a transformer of misery. The problem with the positive activities was that the patient’s ability to engage in valued activity deteriorated. This also explained why the patients with bulbar-onset ALS were happiest with their tube. The tube would be placed when they could still walk and use their arms. Dysphagia and weight loss were their key problems at that time. When these problems were removed, they could spend their time and energy on things they valued, thus turning the tube into a facilitator, with the tube and its management becoming possibly more neutral givens.

Again, this was different for people with limb-onset ALS. To them the tube solved the dysphagia problems, but did not facilitate anything good (when people could not move or concentrate), or gave rise to undesirable events (waiting for the nurse all day). By the time they opted for the tube they could be completely bedridden and close to their death. This is also striking in the sample of patients in the study by Stavroulakis and colleagues (2014): 27 patients volunteered to be interviewed about their tube 3 months after placement. Five of them died before the interview could be conducted, and the condition of eight patients had deteriorated so severely that they had to withdraw. It is a small sample, but significant in that nearly half of the patients dropped out within 3 months after placement. It points to the urgency to consider who could benefit from a feeding tube, or not.

How could the tubes’ identities be so changeable and different for different people? The feeding tube took its shape in the situation in which it was put to use. In itself, the feeding tube did not do much. Its effects and workings emerged in interaction with many variables: the condition of the patient, the availability of informal carers, the required frequency of feeding, the time of placement in relation to their trajectory, and so on. The feeding tube can best be understood not as an intervention that causes ‘impacts on quality of life’, but as a technology or prosthesis that may bring different qualities and appreciations that may shift over time, depending on the way it can or cannot be made to fit in with the patient’s possibility and values.

### Re-visting the Disability Paradox and Response Shift

We can now re-interpret Albrecht and DeVlieger’s (1999) explanation of the ‘disability paradox’ and show why it is incorrect. Rather than balancing goods and bads, as these authors suggest people do to provide an estimation of their quality of life, we saw that people answer questions about their quality of life by interpreting quality in a normative way: they report the *good* things they still experience. Moreover, they do not observe their lives by comparing the situation of their being disabled to the situation before—it there ever was one. Their handicaps have become the ‘facts of life’, the givens that set the stage on which quality may be experienced or not. The ‘bads’ mentioned in de quotes in Albrecht’s and DeVlieger’s paper are all general responses to treatments, like constant pain fatigue and and inability to communicate (p. 984). People do not ‘balance’ these ‘bads’ so that these can be ‘factors that influence quality of life’. Rather, these bads directly interfere with the possibility to have positive experiences at all.

When this analysis is correct, it is hazardous to take ‘functioning’ as an indicator of quality of life. Of course one would not want a treatment to have the effect of reducing peoples’ functioning. This is, however, better understood as an effect on health rather than as a matter of normative quality (experience of a good life), as the latter is not correlated with functioning in a straightforward way. One can indeed be disabled and lead a fulfilling life (Hoppe 2013). When quality is to be understood as relating to what people value and how they evaluate their lives, physical functioning in itself does not influence that in an unequivocal way.

Our findings also add to the understanding of response shift, or the observation that people may judge the same situation differently over time. As we showed, not only peoples’ standards may change, but quality of life cannot be well understood when not taking actual events and processes into account. The tube may turn from a scary symbol of deterioration into an actual solution to a real problem; handicaps may become givens rather than deviations and undesirable states. The emergence of actual events (physiological reactions, functioning) and their appreciations (values, context) are related in complex ways. Now these are studied separately by either biomedical researchers, or social scientists. An ethnographic approach that takes both events and their meanings and contexts into account, may help bridge this gap.

### Measuring Quality

What does this imply for research that does measure ‘the impact of the feeding tube on quality of life’? We could signal some problems inherent to research into quality of life. A first problem was that for ethical reasons, most prominently the emotional weight patients attach to the feeding tube, a randomized trial under standardized conditions is not feasible or desirable. The failed trial described in the introduction illustrated this. The patients did not consent to handing over control about the timing of the placement of the tube, which they saw as a major intervention they’d rather avoid. The different effects for different patients needed to be weighed for each individual.

The second problem with quantifying quality of life in relation to the feeding tube is that the feeding tube’s shifting identities and the process character of quality would be hard to uncover through quantitative studies. The feeding tube forms a complex and unstable intervention, as its effects depend on where and when it is put to work (see also Warrren and Manderson 2013; Warren et al. 2009). Measuring quality over time, from anticipation until death, might be a way to catch some of this process character.

The third, and most fundamental problem is that measurements of effects, be that on quality or health outcomes, presupposes that both peoples’ physiology as well as their values are distributed like in a Gaussian curve. This postulates a general response to treatment, modelled to a shared human physiology where human bodies respond comparably, if gradually different. An example is a generally ‘increased fatigue’ as a reaction to a treatment that may be more or less present in more or less patients in the sample. Individual differences are filtered out by calculating average responses. If, say, five people are not happy with their tube because it merely changed the nature of their misery, whereas ten people are happy because the tube allowed them to do meaningful things, this would lead to a slight positive effect. Differences like the appreciation disfigurement by the feeding tube that are crucial to some, yet trivial to others, would be aggregated in an average score. This would become visible through large standard deviations and indecisive overall outcomes, as is the situation today.^4^

The model of a general physiological response that is normally distributed (as a Gauss curve) does not fit ‘matters of taste’ or value, and in our case also not the actual changes in life. The activities an intervention would allow for or hinder may differ between individuals. The presence or absence of a concern about disfiguring effects is either present or absent. Both effects are erratically distributed through their dependence on context. When a population is grouped together because they share the physiology of the same disease, this does not mean the effects on their lives are the same, or that the research subjects share same values and appreciations. ‘Matters of taste’ are not general physiological responses, but are unevenly distributed, as they depend on context, values, cultural preferences, and so on. What is crucial for the quality of life of one person (e.g. an intact body) may invalidate the life of the next (who fears choking to death), or horrify the outsider who is not (yet) confronted with problems that demand drastic solutions (the freshly diagnosed). Prosthetic and technological aids are likely candidates for this category of interventions that are shaped in time and interaction with their users (Winance 2006; Pols 2012; Hoogsteyns and Van der Horst 2013), but also local circumstances can make the difference. Research in medical anthropology is witness to this. When disturbed sleep is caused by local spirits, people will not take sleep medication, as these will weaken their strength to fight the spirits (Bonelli 2013). These pills will hence have no effect.

The limits of a model of a postulated generalised physiological response demands more research, also within western biomedical traditions. For example: ‘personalised medicine’, is a very different biomedical model to predict individual’s responses to treatment, by relating these to the particular genetic make-up of an individual that differs qualitatively from that of another.

## To Conclude

This study could only lift a tip of the veil that obscures what is measured through quality of life research. We used the very specific case of ALS, the very specific technology of the feeding tube, and we could not access all questionnaires used to asses quality of life in this area. However, our study shows that the search for evidence that a treatment has a positive effect on survival as well as quality should be handled with caution. Questions should be asked whether measuring generalised responses is appropriate, or when we need more fine-grained methods to uncover different sets of generalities that emerge in a population. This question could not emerge in research traditions that study events and interpretations separately. Our suggestion is that we need fine-tuned explorations of what a particular treatment could mean for different patients, both in terms of the characteristics a treatment may have, and in terms of the values people attach to these changes, particularly for complex interventions that gain their effects in relation to context. Different types of generalities may be discovered, generalities that exist as a common response, but is not the same for everyone. Examples were some peoples’ heartfelt concern with the sensuous changes of their body next to others’ indifference to this. These are clinically relevant responses that count for different subgroups of people. Clusters of these types of generalities may then be quantified to describe percentages of people that find them important.

This type of research would do well to dismiss the divide between the study of physiological events versus peoples’ interpretations. For example, we learnt that patients with limb-onset ALS are the most vulnerable to misery transformation by postponing placement or opting for a tube at the end of their trajectory. This provides them with a very unpleasant operation without much to gain from it. On the other hand, attention for aesthetic concerns and solutions, such as ways of covering the tube in situations where others could see it, could enhance the acceptability of the feeding tube for those who might benefit from it. Characteristics and their appreciations are best studied together if one wants to learn about patients’ valuings of treatment.

## Footnotes

1 See for an early feminist discussion on the pros and cons of the use of randomised controlled trials as a means to promote health: Oakley 1990, and for more recent discussions e.g.: Dehue 2002; Timmermans and Berg 2003; Healey 2006).

2 But see for a critical overview of the meanings of quality of life in quantitative science e.g. Thomas, Gill and Feinstein 1994; Moreira 2012; Warrren and Manderson 2013. .

3 An example is Claire Wendland’s (2007), that shows how a cesarian section is not seen as a ‘health consequence’ of treatment because it is intentionally created.

4 It is also likely that people with aesthetic concerns would be underrepresented in the trial, as they may opt to not get a tube. .
